# Annual Meeting of the American Medical Association

**Published:** 1866-07

**Authors:** 


### Miscellaneous.

### ANNUAL MEETING OF THE AMERICAN MEDICAL ASSOCIATION.

### Third Day, May 3d.

The Association was called to order at 9 A. M., by the President, after which the announcement of the members of the Committee to memoralize Congress on the publication of the surgical history of the war was made.

Dr. C. C. Cox, of the Committee on Necrology, reported progress, and on motion of Dr. Hibbard, permission was given the reporter to send the report, when ready, to the Committee on publication.

*The Death of Prof. Joseph M. Smith, of New York.*—Dr. Alfred C. Post offered the following, which was unanimously adopted:

"*Resolved*, That the Association has heard with sincere regret of the death of its late distinguished member, Joseph M. Smith, M. D., of New York.

"*Resolved*, That we cherish his memory as that of a learned and skillful cultivator of medical science, an able and successful teacher and writer, an upright and honorable man, and a patriotic and public-spirited citizen.

"*Resolved*, That the Secretary communicate to the family of the deceased an expression of our sympathy with them in their bereavement."

Dr. C. A. Lee rose to speak to these resolutions, which he did with much feeling. He hardly thought that it was necessary to say anything in regard to the life or character of such an excellent and well beloved man, but as he had been intimately acquainted with him for over thirty years, he did not think it out of place for him to say a few words. After referring in an appropriate manner to his acquaintance with the deceased, he remarked "that a more pure, upright and conscientious man I never knew, particularly with reference to his intercourse with medical men. When I think of the great loss we have sustained in him, I am at a loss to express myself."

Dr. J. S. King, of Natchez, Miss., forwarded a communication to the Association, stating that he was engaged in the compilation of the mortuary and similar statistics of the principal cities and towns of the country, and requesting that physicians would transmit to him such information upon those subjects as they could gather in their respective localities.

The Secretary read a communication from the Dubuque (Iowa) Medical Society, requesting the erasure of the name of Dr. Asa Horr.

On motion of Dr. Jewell, the request was granted.

Dr. Mayburry, on behalf of the Committee on Publication, to whom Dr. Toner's resolutions were referred, reported the following as a substitute, which, on motion, was adopted:

"*Resolved*, That instead of yearly re-printing the list of the members of the American Medical Association, the Committee on Publication be instructed to prepare and print with the Transactions, an alphabetical catalogue tri-ennially, containing a complete list of the permanent members, with their names in full, designating their residences, the year of their admission, the offices they may have held in the Association, and in case of death or resignation, the date thereof."

Dr. Mayburry also presented the following, which, on motion, was referred to the Committee on Ethics:

"*Whereas*, Medical organizations, such as National, State and County Societies, are believed to be absolutely necessary to preserve the honor of the medical profession, and to keep alive social and fraternal feelings among the members thereof, as well as an important means of promoting medical knowledge and elevating the character of the profession; therefore,

"*Resolved*, That it is with sincere regret that we, the members of the Montgomery County Medical Society of Pennsylvania, learn that some honorable members of the faculties of our medical colleges in Philadelphia and elsewhere, have kept aloof from the County Societies on which rest both State and National organizations, thus ranging themselves on the side of those whose unprofessional conduct or low standard of medical attainment, or disregard of medical etiquette, prohibits them from membership in those societies.

"*Resolved*, That as graduates of the University of Pennsylvania, Jefferson Medical College and Pennsylvania Medical College, we have a high regard for the teachers of those institutions, and feel that they owe it to the profession and to our Alma Maters to give their hearty support to medical organizations in general, and especially to the County and State Medical Societies.

"*Resolved*, That although colleges are entitled to representation in the American Medical Association by one or more of their Professors, we are decidedly opposed to any college or any other medical organization being represented by a Professor who is not a member of a County Society.

"*Resolved*, That the Corresponding Secretary of this Society be instructed to report these proceedings to the Philadelphia County Medical Society, and that our delegate be charged to lay them before the American Medical Association at the coming meeting to be held in Baltimore on the first day of May next, as well as before the Medical Society of the State of Pennsylvania at its next meeting, to be held at Kingston, Luzerne county, on the thirteenth day of June ensuing."

W. P. Robinson,
President Montgomery Co. (Pa.) Med. Society.
E. Smyser, Cor. Sec'y.

*The Report of the Committee on Ethics—Specialties in Medicine.*—Dr. Worthington Hooker offered the majority report, and in the main took the ground adverse to exclusive specialties. He divided up the subject into exclusive and partial specialties. In reference to exclusive specialism, he maintained that local affections were apt to be unduly estimated, to the exclusion, perhaps, of other parts of the system that were of more importance in the production of a particular disease; that diseases cured by a specialty are magnified in their importance; that specialists too frequently undervalue the treatment of diseases by the general practitioner; that there is a temptation to employ undue measures to obtain notoriety; and that he is further tempted to charge unduly large fees. The field of medical practice was so large that the profession was always willing to seek advice from those who had devoted attention to particular subjects; but this should not encourage exclusive specialism. The specialty should be a natural outgrowth from the general practice, and should never be separated from it. If this were so, a full, frank and free intercourse would be had between the specialits and general practitioners. The means availed of by the specialists to bring this fact before the public should be ordinary, and not extraordinary. There should be neither advertisements nor puffs in the newspapers. The professor in a school has been chosen for it by those who are competent to discuss his merits for that position; if he were by himself to place before the public the fact that he is specially skilled in the branch taught by him, he would come under this censure.

The report was well drawn up, and claimed the undivided attention of the members.

Dr. Kennedy, of New York, followed with a minority report, stating that he would read it in the absence of the writer. The writer believes that the whole tendency in every department of science is towards specialties. Science has been advanced during the last century by this course. Recently this tendency has shown itself in the persons of certain practitioners who resign all general practice, and confine themselves to the specific department they have chosen. No association can object to the advertisement in such cases, unless it is of a mountebank character. The report was signed by H. I. Bowditch.

The subject was then discussed by Drs. H. R. Storer, of Boston, Worthington Hooker, of New Haven, and others; but the hour of eleven having arrived, Dr. W. Marsden, of Quebec, was introduced, and proceeded to address the Convention on the subject of cholera connected with quarantine.

*Cholera and Quarantine.*—Dr. Marsden, of Quebec, according to previous appointment, made some remarks upon cholera. He commenced by stating his belief in the communicability of cholera, and the efficiency of a rigid quarantine. He has witnessed the first case that occured on the American continent, and since that time has given much attention to the study of the disease. He was now convinced that every case of cholera could be traced to infection, and that the proper soil for the propagation of the disease was found in filth and the neglect of the ordinary sanitary precautions. He believes that all clothing from patients suffering from the disease should be distroyed, and thus be prevented from spreading the disease. He believed that isolation would prevent the appearance of the disease in any community, and related an instance in point which had made such a strong impression upon him that he was caused to think first of his plan of quarantine. It seems that a schoolmistress, in a locality where cholera threatened to make its appearance, consulted the doctor on the best course to pursue. He advised her, as soon as the disease should appear, to isolate the school from the rest of the town, by closing her gates and doors. This was done, and not a single case of cholera occurred within the walls. Dr Marsden next gave the members a detailed account of his system of quarantine.

"1. The cholera quarantine station shall be divided into three separate and distinct sections or departments.

"2. Each of these three sections or departments shall be isolated and seperated from the others by a *cordon* or portion of neutral ground of not less than one hundred feet wide.

"*a.* One of these sections or departments shall be appropriated to the use of the sick, and shall be the hospital department.

"*b.* The next or central section or department shall be devoted to the use of passengers not having had cholera, but from infected vessels.

"*c.* And the third or healthy section or department shall be appropriated to the use of the healthy, who have been removed from the central department, after having performed quarantine there.

"*A.* In the first section or department there shall be three separate and distinct hospitals, besides a convalescent shed or hospital.

"*a.* The one for confirmed cases of cholera to be called the *cholera hospital.*

"*b.* Another for cases of choleraic diarrhœa, or other premonitory symytoms of cholera, to be called the *hospital for cholerine.*

"*c.* The third for all other diseases not cholera or cholerine, but coming from on board infected vessels, or vessels having had cases of cholera on board, to be called the *general hospital.*

"*B.* The next or central section or department, shall be the primary quarantine department, and shall be appropriated to all persons who are not sick, but come from vessels having had cholera on board, and wherein every case on landing shall undergo inspection, washing, cleansing and purifying, both of persons and personal effects. There a quarantine of four days shall be performed, at the end of which period of time all such persons as continue in sound health shall be removed to the final quarantine department, and any that may fall sick or be threatened with sickness during the four days of probation, shall, as soon as detected, be removed to the proper hospital, in the hospital department. There also the healthy inmates shall be removed daily to a new locality, thus occupying four different habitations during their sojourn.

"*C.* The third, or healthy department, shall be the final department, and shall be for all cases coming from the primary quarantine department, after having been cleansed, washed and disinfected, and after having undergone the four days' quarantine; and here a further quarantine of six days shall be performed, (excepting cases coming from the convalescent hospital or shed, hereinafter provided for,) making in all ten days of quarantine, when all persons continuing healthy shall be discharged from quarantine, and be removed from the station. If any premonitory symptoms or other cases of sickness occur in this department during the six days of quarantine, they shall, as soon as discovered, be removed to the proper hospital, in the hospital department.

"No communication shall take place with the hospital department, except through the central or primary quarantine department, for which purpose a passage, unfrequented by the persons undergoing quarantine, shall be set apart and reserved."

Dr. Lee moved the thanks of the Association to Dr. Marsden for his interesting and practical address, and the request of the body that he furnish it with a digest of his communication.

Dr. Bond amended, that those papers accompanying the lecture be commended to the city authorities, and the authorities having such matters in charge throughout the country, for their action.

Dr. Jewell thought the matter should be further investigated, and moved its reference to the Section on Hygiene, to meet that afternoon.

The special business of the day was suspended to allow the Committee on Nominations to report.

*The Officers for* 1866–7.—*President*—H. F. Askee, Delaware.

*Vice Presidents*—W. K. Bowling, Tenn.; J. C. Hughes, Iowa; H. I. Bowditch, Mass.; Thomas C. Brinsmade, New York.

*Permanent Secretary*—William B. Atkinson, Pennsylvania.

*Treasurer*—Casper Wister, Pennsylvania.

*Assistant Secretary*—W. W. Dawson, Cincinnati.

*Committee of Arrangements*—Drs. John A. Murphy, James Graham, R. R. McIllvaine, J. P. Walker, —— Unsicker, William T. Brown, William B. Done, Cincinnati.

*Committee on Medical Education*—Drs. S. D. Gross, D. F. Condie, John Bell, H. J. Bigelow, Charles A. Pope.

*Committee on Prize Essays*—Drs. Francis Donelson, Md.; Josiah Simpson, U. S. A.; C. C. Cox, Edward Warren, W. C. Van Bibber.

*Committee on Publication*—Continued.

*Committee on Medical Literature*—Drs. A. C. Post, Jas. Anderson, H. D. Noyes, T. G. Thomas, Stephen Smith, all of New York.

*Committee on American Medical Necrology*—Continued, with the additions: Dr. Wood, Delaware, substituted for Dr. Couper; John L. Callender, in place of Dr. Bowling, Tenn.; John Blaine, in place of William Pearson, N. J. The following were added:—Drs. R. D. Arnold, Georgia; Lopez, Alabama; G. Dowell, Texas.

*Committee on Climatology and Epidemics*—Continued, with the additions: H. Jones, in place of C. L. Allen, Vt. The following were added to the committee: Drs. U. Harris, Ga.; G. Engelman, Mo.; R. Miller, Ala.; Fenner, La.; G. Dowell, Texas.

All special committees are to be selected by the sections to which the subjects relate.

*The next place of Meeting.*—The place recommended for the next annual meeting of the Association is Cincinnati, Ohio, on the first Tuesday in May.

On motion of Dr. Ordway, of Boston, the report of the committee was adopted.

On motion, the Association went into a committee of the whole to discuss the resolution offered by Dr. Hibberd, having reference to extending the time for the course of study in the different medical colleges.

The whole matter was earnestly discussed by Drs. D. H. Storer, Worthington Hooker, Wright of Ohio, Davis of Ill., and others, and resulted in the passage of the following resolution, offered by the last gentleman:

"*Resolved*, That the Association most earnestly request the medical colleges of the country to hold a convention for thoroughly revising the whole system of medical college instruction for the purpose of establishing more uniformity of time, and a more systematic course of instruction for the whole."

The report of the Committee of the Whole was adopted, and a committee consisting of Drs. Davis, W. Hooker, S. D. Gross, M. B. Wright, and Shattuck, was appointed.

Dr. C. C. Cox read the report "On Rank in the Army," which was referred to the Committee on Publication.

Dr. Cox then offered the following, which was adopted:

"*Resolved*, That the President of this Association bring before the notice of the Military Committees of both Houses of Congress, at as early a period as possible, the present status of medical men in the military service of the United States, and urge upon them that in the army medical bills, under consideration of Congress, the interests of the medical profession shall be so regarded that the medical staff in the service shall, numerically considered, receive the same rank and command as officers in other staffs of the army are justly entitled to."

The committee appointed to act on the foregoing resolution were Drs. D. H. Storer, C. C. Cox, T. Antisell, W. P. Johnson, and C. L. Allen.

On motion of Dr. Cox, the following members, by invitation, were elected: W. D. Stewart, Va.; W. S. Forward, H. W. Stump, and J. L. Chaplain.

A committee was appointed on the subject of Fracture of the Spine, of which Dr. Brown-Séquard was made chairman.

On motion, Drs. A. C. Post, T. Antisell, and J. L. Atlee were added to complete the Committee on Medical Ethics.

*Specialties.*—On motion, the report of the Committee on Ethics, which had been laid on the table, was called up.

On motion of Dr. Toner, the resolution attached to the minority report was omitted, and the reports were both adopted.

A motion to reconsider next prevailed, and the resolution was added to the minority report and referred as before.

Dr. Homberger, of N. Y., made a request to offer a personal explanation, which, after considerable discussion, confusion and sensational speaking, was granted.

On motion of Dr. Sayre, it was agreed to hold an adjourned meeting at 5 P. M., to discuss the subject of cholera.

A communication from Dr. McGee, "On Periosteal Flap Amputations," and one from Dr. Elsberg, N. Y., "On Diagnosis of Diseases of the Larynx," received, and both referred to the Section on Surgery.

The Association then adjourned until 5 P. M.

THIRD DAY—AFTERNOON SESSION, MAY 4.—At 5 P. M., according to previous adjournment, the Association met, and after being called to order, resolved itself into a Committee of the Whole, choosing Dr. Davis as Chairman.

The subject for discussion as previously announced, was—

*Cholera.*—Dr. Sayre, of New York, opened the discussion. He considered that the disease could not reach here unless it was brought here; that it could not be generated here. It multiplies its ravages when filth and uncleanliness abound, and is generated in a sandy, level country, beneath a temperature of 128 degrees. There the decomposing animal and vegetable substances originate this peculiar poison. He believed that it accompanied the individual, and that it did not travel by atmospheric power. He thought that the government was responsible for permitting the disease to get into the land. A rigid, proper quarantine, universally adopted by the General Government in combination with the British Provinces, would, in his opinion, prevent its admission to our continent. We had no quarantine, rightly considered. The disease in 1849 did not originate in Baxter street, New York, but took its origin from an infected person who escaped from quarantine. The cabin passengers escape because the disease has not traveled two hundred feet nor ten feet from the steerage to the cabin. He remarked that he did not believe in mysteries, but wished to understand facts in his own way. If the valuable information that he had received from Dr. Marsden were put into practical application by the General Government, he believed that millions of money and millions of lives would be saved.

Dr. Linton protested against the doctrines advanced that morning and evening. We had medical journals through which we could discuss this subject a long time before the cholera would get here, and a long time before quarantine could prevent its getting here. "Who can believe that cholera could have been prevented from coming here in 1849? I do not believe it is any more contagious than intermittent fever. I am certain that nine-tenths of the physicians of this country are convinced of this fact. I say to the citizens of New York, Baltimore, and Canada, you may have no fears of the cholera. If it comes, it will arise in your midst. Cholera is not a disease!!" He did not believe that there was any truth in the doctrine of contagion. "Cholera breaks out in ships after they are six weeks at sea. I saw a case in St. Louis two months ago. Where did the Asiatics get it from?"

Dr. A. N. Bell, of Brooklyn, N. Y., thought the facts of Dr. Marsden inconsistent with the results of observation. Dr. M. had traced it first from a brig in Liverpool. He did not say that cholera existed in Liverpool at the time. Dr. B. believed cholera could be traced to various places other than Asia. "If cholera is contagious, it takes various roundabout ways of making short journeys. It took an exceedingly roundabout way to the principal cities of Europe. Of the present epidemic, it is said the Mecca pilgrims first had cholera. The evidences I have collected are against strict quarantine. The passengers of the Atlanta were detained at quarantine; no cases occurred among the well passengers after they left the ship. Of all the things likely to originate cholera, none are equal to a crowded, filthy ship. None of the passengers or things of the Atlanta were taken to Ward's Island Hospital. I would protest against the endorsement of any restrictions against persons as advised by Dr. Marsden. The detention of well persons can never protect us from any disease. Our protection is in our clean houses, for cholera often leaps over healthy residences. The action of the health officers at the New York quarantine has been fatal to well persons, and has tended to ward off investigation of the places where cholera originated."

Dr. John L. Atlee, of Pa., said that it was difficult to know the facts in large commercial cities. "There are a thousand avenues to such cities as New York and Boston; but in the inland districts we are more likely to reach a better observation of facts. In 1832 I was in the midst of cholera at Lancaster County Hospital, Pennsylvania. I believed that cholera and yellow fever were diseases independent of any idiomiasmatic conditions of the atmosphere. In July or August, 1854, a certain peculiar condition of the atmosphere existed. The water of the Susquehanna was very low, and the water of the basin very filthy, yet there was no cholera. There were, however, some cases of bilious and intermittent fevers. One day a car of emigrants came from Philadelphia to Columbia; two or three of the passengers were ill, and were put upon the platform. Four gentlemen seeing them there at the point of death, conveyed them to a shed. In the next twenty-four or forty-eight hours not one of them was living. In two or three days the cholera prevailed in Columbia. In the Lancaster County Hospital the winds were from the south. We had no cholera. A few days after the cholera broke out in Columbia, an emigrant reached there afflicted with cholerine. Shortly after, two or three cases of cholera existed. The same train conveyed the cholera to Pittsburgh. Passengers came to the vicinity of Lancaster, at a place called Paradise. Their effects were sent to Lancaster, in a high and healthy location. The relative who washed the clothes died of cholera. It is a contagious disease. Why did it not spread? Why did not small-pox spread? There is an atmospheric condition favorable to the development of the disease. The result of observations in Sweden was that it had been conveyed there by the clothes of sailors. I think Dr. Marsden is right and Dr. Sayre is right, and our friends in Philadelphia must come to the same conclusion if they wish to preserve that metropolis from the ravages of the cholera."

Dr. Sayre said the quarantine law of New York, as now enforced, is a disgrace to civilization. Dr. Carnochan, himself, and others saw the cases on Ward's Island, and they all came to the conclusion that they were not cases of cholera.

Dr. A. N. Bell remarked that Dr. George Ford insisted that the Ward's Island cases he treated were those of cholera.

Dr. Sayre then quoted from Dr. Ford's official statement in the annual report of the Commissioners of Emigration, in which he (Dr. F ) stated on page 52, that those "twenty-seven deaths were caused by *diarrhœa* and dysentery." This was the *official* statement of Dr. Ford.

Dr. Marsden said that cholera followed human travel. He adduced other facts to demonstrate its contagious character. It is infectious in person and personal effects. He urged the necessity of guarding against any communication between the infected and the well. Equanimity, cleanliness and temperance were the three great adjuncts to the quarantine.

Dr. Jewell, of Pa., said: "I have been charged with disseminating cholera. I have done all I could to prevent its entrance to Philadelphia. Cleanliness and ventilation will do much to that end. We have been engaged at that during the past winter. I do not believe in quarantining healthy people. That would be disseminating the disease by giving it to the well persons on vessels where cholera existed. We had the epidemic in the summer of 1849 in Philadelphia. It began in four different portions of the city. The first case was at Richmond, the second at Eighth and Spring Garden streets, the third in Moyamensing. These were all in the center of the city, except at Richmond, and remote from the Delaware. The filth produced the disease in Richmond and along the Delaware. In 1832 the first case was on the Schuylkill, in a canal boat that come down from the upland country. There had been no foreign arrival in Philadelphia. It came from a poisoned atmosphere. In 1849 no fles were living. In Wheeling the birds died. The doctrine of contagion is dangerous, and will deprive the sick of assistance. Small-pox does spread, and if we had not vaccination it would spread more than it does. Contagion and infection are distinct. Contagion is the principle communicating the disease from one person to another. It is not so with cholera. There were no cases of contagion in 1832 or 1849. No vessels arrived with cholera on board. They may have arrived after the disease appeared. I am sorry the resolution was introduced. Next year we shall be better prepared to test the value of Dr. Marsden's information. The poison of cholera will increase rapidly by contact with filth. It is only by purification of the city that cholera can be prevented."

Dr. Lee followed with some brief remarks sustaining the views of Dr. Marsden, and maintaining that it was contagious under certain circumstances. Certain neighborhoods of a very filthy character were not attacked until emigrants came there.

The Committee of the Whole rose, and the Association adjourned without further action.

*Entertainment by the Corporate Authorities.*—The corporate authorities of the city gave an entertainment to the members this evening, at which were present all the notabilities of the city, including the principal officers and members of the City Councils. The entertainment was prepared in the most generous and munificent manner, and reflected infinite credit on the donors. Between four and five hundred gentlemen were present. The supper was called at nine o'clock. A band of music, stationed in the gallery, initiated the occasion with an appropriate air, and at intervals in the course of the evening performed all the national hymns and songs. After discussing the substantials of the bill of fare, the customary toasts were given by Dr. J. Faris Moore, toast-master, and suitably and eloquently responded to by gentlemen of the municipal government of the city, and the officers and members of the Association, the attentions and laudations of the great assemblage being specially directed to the eloquent response made by Dr. N. Pinckney to the toast, "The Navy of the United States and its medical corps." Other toasts were equally well received, and the interest of the supper was sustained until a late hour in the evening.

Fourth Day, May 4.—The Association was called to order at the appointed time, 9 A. M., by the President, after which the minutes of the previous sessions were read by the Permanent Secretary, Dr. W. B. Atkinson, of Philadelphia.

Dr. Cox was, on motion, accredited as a delegate to the foreign societies.

Dr. Garrish, of N. Y., offered the following, which was adopted:

"*Resolved*, That all the members of this Association urge upon the Legislatures of the various States the great importance of making it compulsory that all marriages, births and deaths be registered."

*Medical Rank in the Navy.*—The Naval Committee appointed at the last meeting of the Association having failed to report upon the subject of naval medical rank, it was moved that Surgeons William M. Wood, Ninian Pinckney and David Harlan, U. S. N., be appointed a committee to report upon the subject at the next meeting of the Association. Adopted.

Various amendments were next brought up and laid upon the table.

The reports of the various sections were then in turn called for and adopted.

Dr. Holton, of Vt., offered the following, which was unanimously adopted:

"*Whereas*, The author of the Essay, Dr. H. R. Storer, to whom the prize of $100 from this Association was awarded in 1865, refused to receive the amount thus awarded, consequently increasing the resources of the Association to that amount; therefore

"*Resolved*, That the thanks of this Association are hereby tendered to Dr. H. R. Storer for this display of liberality."

The Committee on Ethics appointed to report on the resolutions of the Montgomery (Pa.) Medical Society, recommended a reference of the whole matter to the Medical Society of that State.

Dr. Holton offered the following, which was lost:

"*Resolved*, That at the future meetings of this Association there shall be two general sessions, one in the morning and one in the evening, unless otherwise ordered."

Dr. King, of Pittsburgh, offered the following:

"*Resolved*, That this Association, approving of the system of quarantine proposed by Dr. Marsden, of Canada, as the most effectual means for preventing the introduction of cholera into this country, do earnestly recommend the propriety of its adoption at all our ports of entry, to the favorable consideration of Congress."

The house then on motion, after a little discussion, went into a Committee of the Whole, Dr. Davis being chairman.

Dr. Bell, of Brooklyn, N. Y., was granted the privilege of making a personal explanation of his statements in reference to cases of cholera on Ward's Island, and although he persisted in his original assertion, the Chair declared that the whole matter was, he presumed, well understood by the Association, there being only a different scientific opinion entertained by two different parties.

The resolution of Dr. King was then taken up, and after much discussion,

Dr. J. H. Burge, of Brooklyn, offered the following, which, after eliciting many remarks from Drs. Horton, Storer, Post, Lee, Pinckney, (U. S. N.) Marsden and J. Anderson, (N. Y.,) was on motion laid on the table. The following is the resolution:

"*Resolved*, That this Association appoint a committee of ten to memorialize Congress to the following effect: That whereas, in the opinion of many eminent physicians, the system of quarantine recommended by Dr. Marsden, of Canada, for protecting our country from Asiatic cholera, would prove effective; therefore, *Resolved*, that we earnestly petition the government of the United States to make an immediate and ample appropriation, and take all other necessary measures to test the utility of said system."

The Committee of the Whole then rose and reported accordingly.

The President resumed his seat.

Dr. Cox moved that Dr. J. C. Tucker, of Nevada, be a member by invitation. Adopted.

Dr. Stokes offered the following as the report of the Section on Psychology, which was accepted and referred:

"The Section on Psychology unite in requesting that a committee be appointed to make a report at the next annual meeting on Insanity, and ask that Drs. Isaac Ray, of Providence, R. I.; Clement A. Walker, Boston, Mass.; A. B. Cabaniss, Miss.; W. S. Chipley, Ky.; John Fonerden, Md., be appointed said committee. CLEMENT A. WALKER, *Chairman*.

WM. H. STOKES, *Sec'y*.

The report of the Committee of the Whole in reference to the question of quarantine was then adopted by the Association.

*Death of Prof. D. L. Magugin, of Iowa.*—Dr. Taylor, of Iowa, presented the following:

"*Whereas*, After a long and laborious life devoted to the practice of medical art and promotion of the interests of medical science, Dr. D. L. Magugin, of Iowa, has been called to the final rest of all good men:

"*Resolved*, That the Association, while deeply regretting the loss they have sustained, will ever keep alive the memory of his many virtues and professional worth, and commend the example of his untiring devotion to our common cause.

"*Resolved*, That a copy of these resolutions be furnished his family, with sincere condolence."

Dr. Garrish, of New York, offered the following:

"*Resolved*, That the members of this Association tender their heartfelt thanks to our professional brethren of Baltimore for the liberal, cordial and satisfactory manner in which they have entertained us."

Dr. D. H. Storer offered his report as delegate to the last meeting of Superintendents of American Institutions for the Insane, and presented the following for adoption:

"*Resolved*, That the Association recommend to the several medical and law schools of the country, the establishment of an independent chair of Medical Jurisprudence, to be filled if possible by teachers who have studied both law and medicine; attendance upon one full course of lectures from whom shall be deemed necessary before the medical degree is conferred.

"*Resolved*, That while this Association regrets that the Association of Superintendents of American Asylums for the Insane has not yet thought fit to unite itself more closely with the representative body of American physicians, it still is of opinion that such union is for their mutual and reciprocal advantage, and that it ought to be effected without further delay."

On motion, the above was adopted.

After the transaction of business of minor importance, the Association adjourned *sine die*.

---

